# Molecular docking studies of a-mangostin with oral cancer targets ARRB1, FLNA, CALM3 and HTT

**DOI:** 10.6026/97320630016625

**Published:** 2020-08-31

**Authors:** Janardhanan Sunitha, Jaideep Mahendra, Little Mahendra, Nalini Devaraj

**Affiliations:** 1 MAHER, India; 2 Meenakshi Ammal Dental College and Hospital, India; 3 Maktoum Bin Hamdan Dental University College, India

**Keywords:** Oral Cancer, Alpha mangostin, Molecular Docking

## Abstract

Background and Aim: The genes ARRB1, FLNA, CALM3, and HTT are commonly expressed in oral cancer and have been hypothesized to be involved in the carcinogenic pathway. The present study investigates the inhibitive properties of alpha mangostin on the above gene using Autodock molecular docking tool. Materials and Methods: The structures of the proteins were downloaded from the protein databank with PDB IDs 3HOP, 2F3Z, IZSH and 3IO6F for the genes FLNA, CALM3, ARRB1 and HTT, respectively. Autodock was used for molecular docking of the target proteins with the ligand molecule. Results shows HTT having good inhibition features with the Alpha Mangostin followed by the CALM3, FLNA and finally ARRB1 in the decreasing order. CALM3 gene had the lowest binding energy, which easily bound with the target ligand with greater affinity towards the binding followed by ARRB1, HTT, FLNA in the increasing order of binding energy and decreasing order of binding affinity. CALM3 and HTT were promising targets for anticancer treatment using alpha mangostin. Future exploration of the interaction of alpha mangostin and these genes could delineate the role of alpha mangostin as an anticancer agent.

## Background

Mangosteen or Garcinia mangostana is a fruit that is native to Vietnam, Thailand, Indonesia, Malaysia, and other parts of Southeast Asia [1]. The pericarp of this fruit is used in traditional medicines for the treatment of diarrhoea, dysentery, abdominal pain, and wounds [2]. Xanthone is an active ingredient found in the pericarp of the mangosteen fruit, contributing to its anti-inflammatory, antibacterial, antioxidant, and anticarcinogenic properties. One of the most important natural derivatives of xanthone is alpha mangostin, which is a proven anticancerous agent both in-vitro and in-vivo [3]. Globally, Oral cancer ranks sixth amongst the most commonly occurring cancers. It is also the second most common cancer in men. Despite the advanced treatments options against oral cancer through various modalities like chemotherapy, radiotherapy, and surgery, the mortality remains high [4,5]. These conventional cancer treatments are highy toxic to normal cells leading to severe adverse effects [6]. Treatment of cancer using naturally derived molecules have gained acceptance as these are less destructive than the conventional methods [7].

Carcinogenesis, is a complex, multistep process involving numerous signaling pathways and leading to a quantitative alteration in cell physiology. Carcinogenesis also involves alterations in genes like point mutations, rearrangements, amplifications, and deletions [8]. Conventionally a cell should undergo six or more mutations to get transformed into a malignant cell [9]. Once a cell becomes cancerous it is no longer under the control of regulatory mechanisms within the body. Genetic changes may occur at the level of the protooncogenes resulting in a gain of function. Alternatively, the recessive component could involve the growth inhibitory genes or tumor suppressor gene and results in loss of function.

Previous studies on oral cancer have shown that the four genes namely CALM3, ARRB1, HTT, and FLNA were involved in a carcinogenesis pathway [9]. Among these the CALM3 gene or the protein Calmodulin is a critical molecule that plays an important role in calcium-dependent signaling for various physiological processes within the cell [10]. Beta arrestin1 (ARRB1) is an ubiquitously expressed protein that plays a key role in nuclear transcription in cancer cells [11]. Huntingtin (HTT) is a pervasive scaffold protein, which is seen in a neurological disorder called Huntington's disease (HD). This protein appears to play an important role in the central nervous system in cell division, intracellular transport, and transcriptional regulation. FLNA gene codes for Protein filamin, A which along with actin forms the structural framework or cytoskeleton of the cell [12]. Additionally, this protein has myriad functions like cell signaling, phosphorylation, ion channel regulation, proteolysis, and transcription regulation. Thus mutations in this gene lead to an array of symptoms as it impacts multiple systems [13]. Indian traditional medicines have gained lot of importance in the treatment of cancer especially in its initial stages without any side effects. Alpha mangostin, an extract from the Garcinia mangostana, has shown anticancerous activity. In the present study, the inhibitory role of the alpha mangostin on the genes implicated in Oral cancer namely CALM3, ARRB1, HTT, and FLNA was investigated through molecular docking techniques.

## Methodology

### Protein Preparation:

The structures of the proteins were downloaded using database ID 3HOP, 2F3Z, IZSH and 3IO6F from the major protein database http://www.rcsb.org/pdb/home/home.do as the docking target. Proteins were loaded from the menu using the "Load molecule" option. 'Hydrogens' were added from the 'Edit menu'. The ‘Repair module’ was loaded from the 'File menu' to check for missing atoms and they were corrected. Charges were tested for integral values. The macromolecule file was named 'Pdbqs'.

### Ligand Preparation:

The structure of alpha mangostin was downloaded from pubchem database. The ligand was loaded using the choice of input molecule. The rigid root was procured by selecting the atom from the rigid root selection. The rotatable bonds were defined using the option of rotatable bonds. Write pdbq was used to save the resulting pdbq format, which adds the load parameter to the ligand.

### Protein-ligand interaction using Autodock:

By using the Autodock tools [14,15] (ADT) v1.5.4 and Autodock v4.2 program, the docking analysis was carried out. In order to run the docking, the search grid, extended to the target protein was used and the polar hydrogen was applied to the ligand. Kollman charges were allocated and the parameters for the nuclear solution were added. Polar hydrogen charges of the Gasteiger form were allocated and the non-polar hydrogen was merged with the carbons and the internal degrees of freedom and torsion were formed. During the docking cycle target proteins were held as rigid and ligands allowed traveling freely. Using the blind docking method, the quest was extended to the entire protein. Affinity maps for all atom types were present, as well as electrostatic map, was computed with a grid spacing of 0.375 Å. The Lamarckian Genetic Algorithm was used to search for populations of 150 individuals with a mutation rate of 0.02 over 10 generations. The results were sorted on the basis of the binding energy. Based on the root mean square deviation (RMSD) values, a cluster analysis was performed with reference to the starting geometry. The lowest energy content of the more populated cluster was considered to be the most reliable solution.

## Results and Discussion:

The four genes CALM3, ARRB1, HTT, and FLNA were identified to be involved in a carcinogenic pathway [16] The gene ARRB1 forms a complex which on dissociation, causes activation of RAS like proto-oncogene A(RALA) and regulates the actin cytoskeleton through FLNA. Thus the intracellular signaling pathway is activated by calcium-ion binding with CALM3, which also involves RAL [17]. The effector protein of RALA is FLNA, which in turn stimulates p21-activated kinase 1(PAK1). Activation of PAK1 stimulates actin cytoskeleton reorganization. This enhances the cell migration and facilitates invasion of cancer cells. Additionally, the PAK1 molecules also cause HTT aggregation in the cell, which is toxic to the cell and ultimately leads to cell death. The influx of calcium ions into cells after their stimulation by growth factors is a crucial step in cell proliferation. These calmodulin-regulated pathways, along with the mitochondria and the endoplasmic reticulum, also play a role in apoptosis and cell cycle activation of cyclin-dependent kinases, nucleotide metabolism, and chromosomal reorganization [18]. In tumor cells, there is an alteration of this calmodulin regulated cell regulation and proliferation. The concentration of calmodulin in the nucleus induces the cancer cells to proliferate. This also causes angiogenesis in tumor cells by forming complexes with calcium on being induced by hypoxia [19]. ARRB1is shown to interact with hypoxia-inducible factor (HIF -1A) and stimulate HIF-1A mediated transcription, thereby inducing proliferation of cancer cells. ARRB1 acts as tumor promoter by enhancing glycolysis within the cell and decreasing mitochondrial activity [20].

The role of FLNA gene in cancer seems to be multidimensional and plays an important role in cell signaling. Although there is an overexpression of FLNA in various types of cancer, the effect it has on the cellular functions are varied [21]. FLNA seems to promote cell migration and enhances the invasive potential of cancer cells. Antagonistically it also regulates focal adhesion disassembly, thus suppressing cancer cell migration. Thus it is contended that the FLNA can induce or suppress the migration of cancer cells based on their binding proteins availability. If present in the cytoplasm, it promotes the growth of cancer and its migration, On the contrary, it inhibits the growth of cancer cells if the FLNA is localized to the plasma membrane [22]. In the present study, we explored the role of active ingredient of mangosteen pericarp: alpha mangostin on four genes involved in oral cancer, which together are interlinked to form a pathway. Activities of α mangostin against these four genes were identified through molecular docking analysis. AutoDock software was used to perform docking simulations between α mangostin with four genes involved in oral cancer. The results of the docking between α-mangostin with four genes are presented in Table 1. Out of the four different genes docked, with reference to the RMSD value, HTT gene, which associates with the huntington disease showed a good inhibition with the Alpha Mangostin followed by the CALM3, FLNA and finally ARRB1 in the decreasing order.

**Figure 1:**
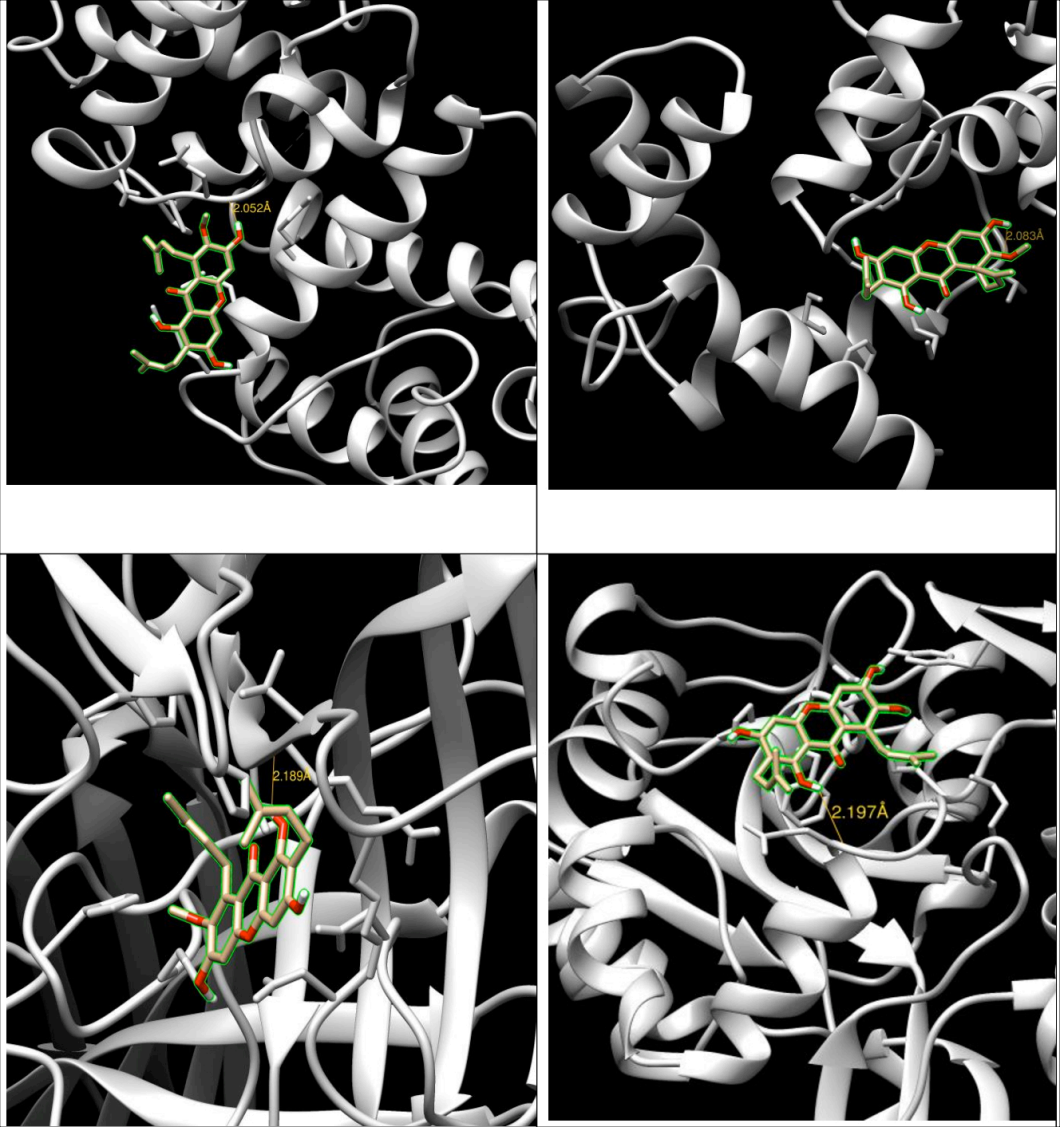
Visualization of Interactions of α-mangostin with the target proteins. The ligand is represented in green chain. (a) Interactions between α-mangostin with 3hop; (b) Interactions between α-mangostin with 2f3z; (c) Interactions between α-mangostin with Izsh; (d) Interactions between α-mangostin with 3io6-HTT

The minimal energy needed to form a complex between the ligand and the receptor suggests an excellent binding affinity. Rather, low energy means that the ligand is buried in the receptor cavity [23]. Results of docking studies showed that all the four genes had minimum binding energy; hence compound α mangostin was bound to the cavity of the receptor and had the capacity to inhibit the activity of these four genes. On considering the binding energy of the Alpha Mangostin against the docked genes, the CALM3 gene had the lowest binding energy, which easily bound with the target ligand with greater affinity towards the binding. This was followed by ARRB1, HTT, FLNA in the increasing order of binding energy and decreasing order of binding affinity. Moreover the ligand alpha mangostin had formed a hydrogen bond with all the proteins (Figure 1). The distance and site of hydrogen bond is mentioned in the Table 1. The distance of the H-bonds was less than three, suggesting the existence of desirable interactions between ligand and receptor. All the four complexes had H-bond distance less than three. So this also indicated that the Alpha Mangostin showed the good activity against these four genes With reference to the RMSD value, HTT gene, which usually associates with the Huntington disease, showed a good inhibition with the Alpha Mangostin followed by CALM3, FLNA and finally ARRB1 in the decreasing order. In a similar study on the mechanism of inhibition of ethanolic extracts of mangosteen pericarp on HeLa cell lines using molecular docking techniques, it was found that the NFκB (Nuclear Factor kappa-light-chain-enhancer of activated B cells) pathway which plays a key role in the production of anti-apoptotic proteins like Bcl-2, Bcl-xl, and BFL1 are influenced by mangosteen. NFκB activation requires several steps either phosphorylation of IκB by IKK or IκB degradation by the proteasome. The former IKK is more likely involved than proteasome [24]. Since the binding energy of two molecules is inversely proportional to binding affinity, RMSD score is directly proportional to binding affinity. Considering both the factors, the most suitable target gene for Alpha mangostin was in the order of CALM3, HTT, ARR1, and FLNA. This study provides an insight regarding the use of alpha mangostin in the treatment of oral cancer and the potential candidate genes.

## Conclusion

Alpha mangostin showed that CALM3 and HTT as CALM3 had the lowest binding energy among the four genes and HTT showed a good inhibition with respect to RMSD value. Data shows that alpha mangostin in inhibiting the oral cancer genes. Furthermore, molecular docking studies in association with the alpha mangostin on the most promising genes CALM 3 and HTT are required, which could pave the way for using this extract in targeted therapy against oral cancer cells.

## Declaration on Publication Ethics:

The authors state that they adhere with COPE guidelines on publishing ethics as described elsewhere at https://publicationethics.org/. The authors also undertake that they are not associated with any other third party (governmental or non-governmental agencies) linking with any form of unethical issues connecting to this publication. The authors also declare that they are not withholding any information that is misleading to the publisher in regard to this article.

The authors are responsible for the content of this article. The Editorial and the publisher has taken reasonable steps to check the content of the article with reference to publishing ethics with adequate peer reviews deposited at PUBLONS.

## Figures and Tables

**Table 1:** The distance and site of hydrogen bonds formed between alpha mangostin and the four proteins.

Proteins Ligand	3hop α-Mangostin	2f3z	1zsh	3io6-HTT
Binding energy	-3.32	-6.36	-4.46	-3.93
Ligand efficiency	-0.11	-0.21	-0.15	-0.13
H acceptor	UNL1: H	UNL1: H	UNL1: H	UNL1: H
H donor	A: GLN240:0	A: THR79:0	A: ARG165:O	A: GLN325:0
Distance	2.052	2.083	2.189	2.197
RMSD Score	51.79	60.8	46.33	88.92
